# Annotations

**Published:** 1907-10-12

**Authors:** 


### Annotations.

### Malta Fever.

The dispute which has unfortunately arisen in connection with the discovery of the cause of Malta Fever and the relation of the disease to the milk of infected goats, has received a notable contribution from Colonel David Bruce, C.B., F.R.S., who was the Chairman of the Malta Fever Commission of the Royal Society. According to Colonel Bruce, the actual observation which led to the discovery of the true nature of the disease was made by Dr. T. Zammit, the Maltese member of the Commission, who made a number of experiments by feeding goats with material containing the *Micrococcus Melitensis*. At Colonel Bruce's suggestion these investigations were continued, and a small herd of goats was purchased for the purpose. Before, however, repeating the feeding experiments Dr. Zammit proceeded to examine the blood of the goats, and then, to his surprise, found that in the great majority of instances the blood gave a reaction characteristic of Malta fever. He at once submitted the facts to Major Horrocks, R.A.M.C., who confirmed the observations, and proceeded to examine the blood for the specific organism. At the same time Dr. Zammit subjected the milk of the goats to a similar search, and in this way was established the facts that the micro-organism was present in the blood and was excreted in the milk of 10 per cent. of the goats of Malta. Another observation of great significance was also made by Major Horrocks, namely, that the disappearance of Malta fever and of the Maltese goats from Gibraltar were coincident events. It is these observations which dealt the death-blow to the previously existing view that Malta fever was due to "an aerial virus from infected soil or drains." The miasma theory has had a long popularity in reference to the causation of disease, but modern observations have seriously curtailed its range.

### The Medical Societies.

The issue of the programmes of the various professional societies intimates the close of the holiday season and the renewal of the scientific and practical activities upon which the progress and advance of medicine depend. The ensuing session will certainly not fall below the standard of former records, and especial interest will attach to it in view of the recent establishment of the Royal Society of Medicine. The clinical section of the new Society has for its President Sir Thomas Barlow, who will deliver an inaugural address on Friday, October 11, at 8.30 p.m.; on the same evening, at eight o'clock, a number of clinical cases will be exhibited. The Medical Society of London is to open its 135th Session on Monday, October 14, with an address from the President, Dr. J. Kingston Fowler, after which a paper will be read by Dr. J. Rose Bradford on "Some Unusual Cases of Diabetes." The Lettsomian lectures will be given by Mr. Charters James Symonds, while the annual oration is to be delivered by Mr. Clinton T. Dent. The programme also includes discussions on "Pneumococcus Infections," to be opened by Professor William Osler; on "The Diagnosis and Treatment of Fractures of the Long Bones," to be introduced by Mr. A. Pearce Gould; and on "The Prognosis and Treatment of Acute Anterior Poliomyelitis," in which Dr. J. Risien Russell will be the first speaker. At the Society for the Study of Disease in Children there is to be a special discussion on Congenital Syphilis, and the Wightman Lecturer is Mr. Watson Cheyne, C.B.; the first clinical meeting is October 18 at 5 p.m. The session of the Medico-Legal Society commences on Tuesday, October 29, when the President, Mr. Justice Walton, will deliver his inaugural address, and Dr. W. H. Willcox will give a demonstration on "The Medico-Legal Importance of Wounds produced by Firearms." Another event of the present month is the Harveian oration at the Royal College of Physicians by Dr. Frederick Taylor, Consulting Physician to Guy's Hospital.

### The Midwives Act.

It is evident from a recent correspondence in the *Times* that the great expectations indulged in by the promoters of this Act are likely to encounter difficulties which were by no means anticipated. After January 1, 1910, the practice of midwifery by unlicensed women will be totally prohibited, and already great anxiety is felt in reference to the possibility of securing an adequate number of women to act as midwives. Though we are told that under the new Act the midwife will be a "professional person," it seems that the rewards offered to the "profession" are so scanty that there is fear lest the number of candidates for these should fall below what are considered to be the requirements of the country. Hence it is declared that the question of the provision of funds, whether public or private, to secure the training and adequate payment of a sufficient number of midwives will have to be resolutely faced. Voluntary contributions to a national fund are suggested as one solution of the difficulty, and a large public meeting to promote the necessary agitation is announced. Another proposal doubts the adequacy of this suggestion, and urges that State aid must be given. Parliament, it is said, has created the difficulty, and must now be called upon to find a solution. Obviously, therefore, the good intentions of those who promoted the Act have led, as such motives often do, to practical disadvantages which it will by no means be easy to remove. There can be but one desire that women of the poorer classes shall receive efficient help during childbirth. But whether the Midwives Act was the best way to obtain this end is open to discussion. In addition, it is a fair question whether a rule of thumb training—and little more than this is possible—can ever qualify a midwife to undertake some of the functions with which she is to be charged. These, in the latest pronouncement, include the prevention of maternal death and infantile blindness, and the "instruction" of the mother and those surrounding her concerning things to be done for her benefit and things to be avoided. Medical practitioners have many chances of judging of the nature of "instructions" conveyed by the amateur or half-trained individual, and so far the teaching has failed to excite professional enthusiasm.

